# Clinical Presentation and Disease Course of 37 Consecutive Cases of Progressive Multifocal Leukoencephalopathy (PML) at a German Tertiary-Care Hospital: A Retrospective Observational Study

**DOI:** 10.3389/fneur.2021.632535

**Published:** 2021-02-04

**Authors:** Lisa M. Graf, Sina C. Rosenkranz, Angelique Hölzemer, Christian Hagel, Einar Goebell, Sabine Jordan, Manuel A. Friese, Marylyn M. Addo, Julian Schulze zur Wiesch, Claudia Beisel

**Affiliations:** ^1^Division of Infectious Disease, I. Department of Internal Medicine, University Medical Center Hamburg-Eppendorf, Hamburg, Germany; ^2^Institute of Neuroimmunology and Multiple Sclerosis, University Medical Center Hamburg-Eppendorf, Hamburg, Germany; ^3^Department of Neurology, University Medical Center Hamburg-Eppendorf, Hamburg, Germany; ^4^German Center for Infection Research (DZIF), Partner Site Hamburg-Lübeck-Borstel-Riems, Hamburg, Germany; ^5^Heinrich Pette Institute, Leibniz Institute for Experimental Virology, Hamburg, Germany; ^6^Institute of Neuropathology, University Medical Center Hamburg-Eppendorf, Hamburg, Germany; ^7^Department of Diagnostic and Interventional Neuroradiology, University Medical Center Hamburg-Eppendorf, Hamburg, Germany; ^8^Department of Tropical Medicine, Bernhard Nocht Institute for Tropical Medicine, Hamburg, Germany

**Keywords:** progressive multifocal leukoencephalopathy (PML), risk factors, PML-directed treatment, survival rate, HIV, JCV

## Abstract

**Background:** Progressive multifocal leukoencephalopathy (PML) caused by JCV is a rare but frequently fatal disease of the central nervous system, usually affecting immunocompromised individuals. Our study aims to expand the data on patient characteristics, diagnosis, clinical course, possible PML-directed treatment, and outcome of patients with PML at a German tertiary-care hospital.

**Methods:**In this single-center observational cohort study, 37 consecutive patients with a confirmed diagnosis of PML seen at the University Medical Center Hamburg-Eppendorf from 2013 until 2019 were retrospectively analyzed by chart review with a special focus on demographics, risk factors, and clinical aspects as well as PML-directed treatment and survival.

**Results:**We identified 37 patients with definite, probable, and possible PML diagnosis. 36 patients (97%) had underlying immunosuppressive disorders such as HIV/AIDS (*n* = 17; 46%), previous treatment with monoclonal antibodies (*n* = 6; 16%), hematological or oncological malignancies (*n* = 6; 16%), sarcoidosis (*n* = 5; 14%), solid organ transplantation (*n* = 1; 3%), and diagnosis of mixed connective tissue disease (*n* = 1; 3%). In only one patient no evident immunocompromised condition was detected (*n* = 1; 3%). Treatment attempts to improve the outcome of PML were reported in 13 patients (*n* = 13; 35%). Twenty seven percent of patients were lost to follow-up (*n* = 10). Twenty four-month survival rate after diagnosis of PML was 56% (*n* = 15).

**Conclusion:** This interdisciplinary retrospective study describes epidemiology, risk factors, clinical course, and treatment trials in patients with PML at a German tertiary-care hospital. Acquired immunosuppression due to HIV-1 constituted the leading cause of PML in this monocenter cohort.

## Introduction

Progressive multifocal leukoencephalopathy (PML) is a severe demyelinating disease of the central nervous system that is caused by reactivation of the JC virus (JCV) in immunocompromised individuals (1). In rare instances, JCV infection is diagnosed in patients with no apparent immune defect or immunosuppression. Primary infection with JCV occurs early on in childhood and stays asymptomatic. In adults, JCV-specific antibodies can be found in over 50% above the age of 20 years (2). In immunocompetent individuals JCV remains latent in kidneys and lymphoid organs. However, in patients with cellular immunosuppression, genomic rearrangements of the viral genome can lead to neurotropic variants with the ability to replicate in glial cells. JCV can then invade the brain and induce a lytic infection of oligodendrocytes (3). Infrequently, PML is the result from of a primary infection with JCV and not always means a reactivation of a latent JCV infection.

Generally, PML is a rare disease and so far there are only a few population-based studies that have mostly investigated incidence rates in specific populations at risk, such as patients with malignancies, HIV infection, solid organ transplantations, or patients with autoimmune disorders receiving immunomodulatory treatment with monoclonal antibodies (mAb) (4–9). Historically, PML has mainly been observed in persons with hematologic malignancies and HIV/AIDS, while more recently PML has increasingly been diagnosed in the context of natalizumab, a mAb used for treatment of relapsing-remitting multiple sclerosis (4, 5, 10, 11). According to the current safety report of the manufacturer of natalizumab (*Biogen*), the number of multiple sclerosis-natalizumab-associated cases of definite PML continues to rise and had reached 839 cases by August 2020, of which 518 occurred in Europe.

The clinical manifestations of PML are diverse since they are linked to the location and extent of damage in the central nervous system. Neurological symptoms can rapidly progress within days. Common symptoms are progressive weakness and alterations in visual acuity, speech, and personality (4). The diagnosis of PML can be made by typical clinical findings, compatible lesions of the white matter on magnetic resonance imaging (MRI) scans, and the detection of JCV DNA in cerebrospinal fluid (CSF) via polymerase chain reaction (PCR) or histological examination of brain biopsies (12, 13).

Although off-license drugs are reported in the treatment of PML (14), the patient outcome depends on the ability to restore immune function in response to JCV. Current treatment strategies aim at restoring immune function to successfully improve survival, in particular in patients with HIV/AIDS. However, the prognosis for the majority of PML patients remains poor (15).

PML associated immune reconstitution inflammatory syndrome (PML-IRIS) is an immunological phenomenon, where PML may develop or worsen despite a recovery of the immune system. The inflammatory process deteriorates symptoms and potentially leads to brain injury. Patients may show imaging signs suggestive of inflammation (16, 17).

PML risk prediction tests are clinically not well established. Sequential JCV antibody tests are currently applied during natalizumab treatment but have low specificity. Recently, genetic risk variants in patients with PML were identified (18), which might help to assess patients at risk, especially in those using immunosuppressant agents.

Here, we present an observational cohort study at a German tertiary-care hospital in northern Germany, to investigate patient characteristics, diagnosis, clinical course, possible PML-directed treatment, and survival of patients with PML.

## Methods

In this retrospective cohort study, we analyzed data of patients with PML, identified by appropriate ICD10 codes using the local patient register. All patients presented consecutively at the University Medical Center Hamburg-Eppendorf, Germany, in the time period from January 2013 until August 2019. The diagnosis of PML was confirmed by a review of the available medical records. Only patients with a definitive, probable, or possible diagnosis of PML were enrolled in this monocenter, retrospective cohort study (13). The center is a tertiary care referral center for HIV/ AIDS and multiple sclerosis therapy in Germany. The center is a University Medical Center in Northern Germany with a catchment area of 4 Million patients with referrals center for HIV/ AIDS and multiple sclerosis as well as Oncology, stem cell and organ transplantation centers as well as Rheumatology department. Patients were referred from specialized neurologists to the multiple sclerosis outpatient and inpatient clinic and from infectious diseases specialists to the HIV medical department and its outpatient center. Also, general practitioners sent patients for further evaluation and treatment from the whole northern part of Germany to both clinics.

### Definitions

**Figure 1:**
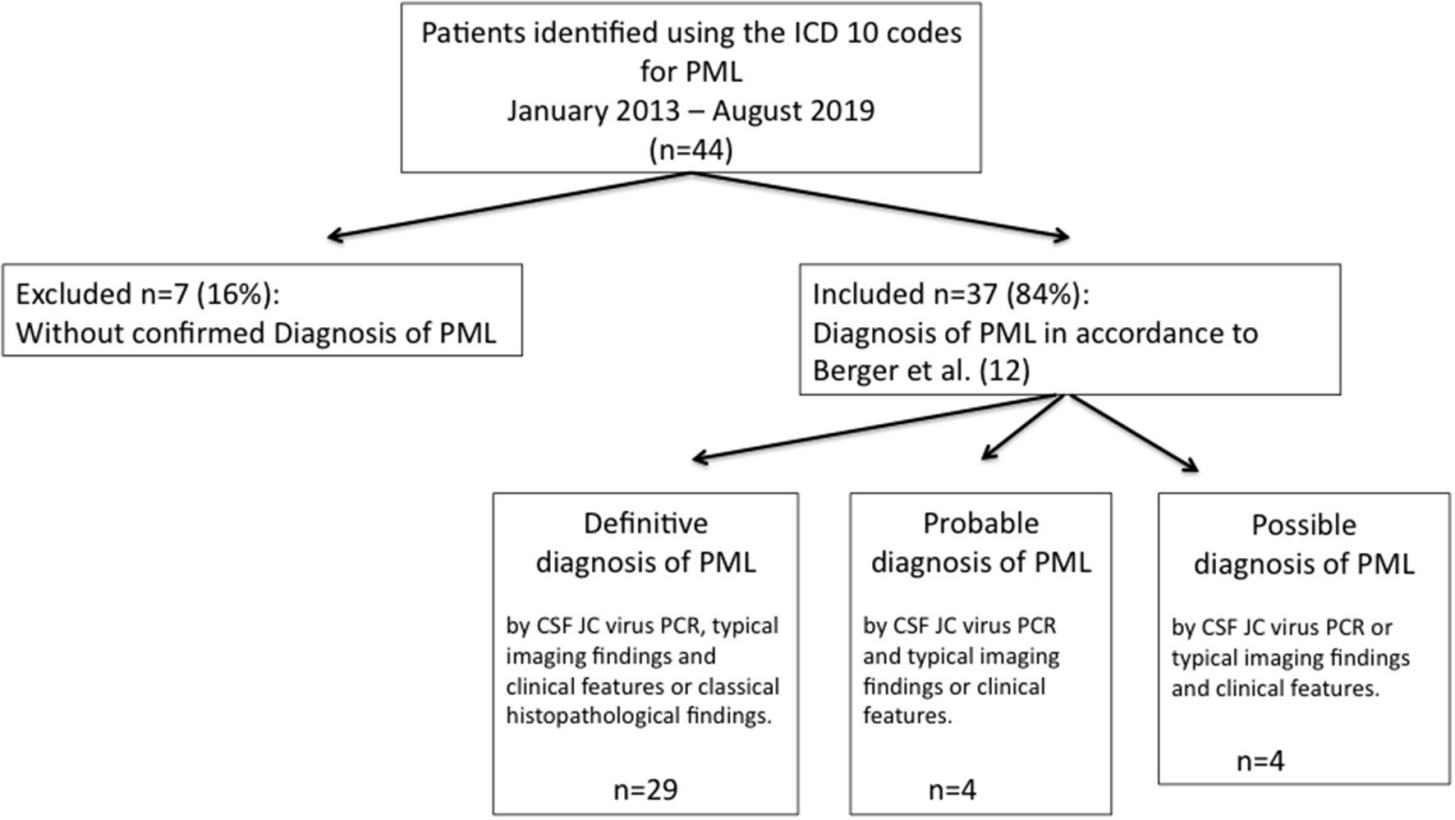
Diagnostic flowchart of patients with diagnosed PML. As underlying immunosuppressive condition patients with possible PML (*n* = 4) all suffered from HIV.

In accordance with the Consensus Statement of the *American Academy of Neurology (ANN) Neuroinfectious disease section* (13) diagnosis of PML was classified as definite, probable, and possible certainty (Figure 1). Survival was assessed at 12 and 24 months after the diagnosis of PML.

### Diagnostics

JCV PCR diagnostic was performed with a commercial assay as recommended by the manufacturer (RealStar JCV Kit, Altona Diagostik, Hamburg, Germany). For the detection of JCV specific DNA, the analytical sensitivity is 1.365 copies/μl [95% confidence interval (CI): 0.568–5.831 copies/μl], according to the user manual.

### Statistical Analysis

Statistical analyses were carried out using SPSS and GraphPad Prism 5 software.

### Ethical Statement

The local ethics committee of the Ä*rztekammer Hamburg* approved the study (WF-179/20).

## Results

### Demographics

Between January 2013 and August 2019, a total of 37 patients with the diagnosis of PML were consecutively identified at the University Medical Center Hamburg-Eppendorf and included in this retrospective monocenter study. At the time of diagnosis the age of the patients ranged from 25 to 81 years, with a median age of 50.5 years. With 27 of 37 patients (73%), there was a higher proportion of male individuals.

### Underlying Conditions and Immunosuppressive Therapy Before the Diagnosis of PML

Detailed information on underlying and immunocompromised conditions is given in Table 1. Leukocyte count was available in 86% of patients (*n* = 32/37) with a median of 5,5/μl (range 1.8–12/μl; norm: 3.8–10/μl). Mild leukopenia was seen in 10 patients, where leukocyte count was available (*n* = 10/32; 31%). CD4 T cell count was available in 24 patients (*n* = 24/37; 65%) with a median of 104/μl (range 17–630/μl; norm: 300–2200/μl).

Table 1Baseline characteristics of patient cohort, underlying conditions and administered immunosuppressive agents prior to diagnosis of PML.

**Table:** 

**Baseline characteristics of patient cohort**			
**All Patients**, *n* (%)	*n* = 37 (100)		
**Age (y)**, median (min./max.)	50.5 (25 / 81)		
**Sex**, male/female (%)	27/10 (73/ 27)		
**Leukocytes** at time of diagnosis			
**Available in number of patients**, *n* (%)	32 (86)		
Median (min./max.)	5.5 (1.8–12)		
**CD4 T cell count** {/μl}at time of diagnosis			
**Available in number of patients**, *n* (%)	24 (77)		
Median (min./max.)	104 (17–630)		
**JCV PCR** {copies/ml} at time of diagnosis			
Median (min./max.)	3,000 (20/ 400 000)		
**Patients with HIV/ AIDS**			
**CD4 T cell count** {/μl}at time of diagnosis			
Median (min./max.)	70 (17–314)		
**CD4/CD8 T cell ratio** at time of diagnosis			
Median (min./max.)	0.12 (0.03–0.24)		
**HIV viral load**			
Median (min./max.)	17,500 (0–2,212,797)

**Table:** 

**Underlying condition**	**Total number of patients** ***n*** **(%)**	**Mortality** ***n*** **(%)**	
**All Patients**	37 (100)	12 (32)	
**HIV/ AIDS^a^**	17/37 (46)	4/17 (24)	
**Malignancies**	6/37 (16)		
Hematological malignancy	5/37 (13)	4/5 (80)	
Solid tumor and hematological malignancy	1/37 (3)	1/1 (100)	
**Autoimmune diseases with chronic immunomodulatory therapy**	6/37 (16)		
Multiple Sclerosis	5/6 (83)	1/5 (20)	
Ulcerative Colitis, PSC, AIH^b^	1/6 (17)	0 (0)	
**Sarcoidosis**	5/37 (13)	1/5 (20)	
**Mixed connective tissue disease**	1/37 (3)	0 (0)	
**Liver transplantation**	1/37 (3)	1/1 (100)	
**No evident immunocompromised condition**	1/37 (3)	0 (0)

**Table:** 

**Immunosuppressive and immunomodulatory agents**	**Total number of patients** ***n*** **(%)**	**Autoimmune diseases with chronic immunomodulatory therapy** ***n*** **(%)**	**Hematological malignancies** ***n*** **(%)**	**Solid tumors, Organ transplants** ***n*** **(%)**	**Sarcoidosis** ***n*** **(%)**	**Mixed connective tissue disease** ***n*** **(%)**
**Natalizumab**	5/37 (13,5)	5/6 (83)	0 (0)	0 (0)	0 (0)	0 (0)
**Allogenic stem cell transplantation**	2/37 (5)	0 (0)	2/6 (33)	0 (0)	0 (0)	0 (0)
**Other immunosuppressive agents**						
Combination chemotherapy^*^	5/37 (13,5)	0 (0)	5/6 (83)	1/2 (50)	0 (0)	0 (0)
Prednisolon only	1/37 (3)	0 (0)	0 (0)	0 (0)	1/5 (3)	0 (0)
Tacrolimus	1/37 (3)	0 (0)	0 (0)	1/2 (50)	0 (0)	0 (0)
Azathioprine	3/37 (8)	1/6 (17)	0 (0)	0 (0)	1/5 (3)	1/1 (100)
Methotrexate	1/37 (3)	0 (0)	0 (0)	0 (0)	1/5 (3)	0 (0)

a *HIV/ AIDS, Human immunodeficiency virus/ Acquired Immune Deficiency Syndrome*.

b *PSC, primary sclerosing cholangitis; AIH, autoimmune Hepatitis*.

* *One patient was diagnosed with both, a solid tumor and a hematological malignancy*.

Forty six percent of patients (*n* = 17/37) suffered from HIV/AIDS. Fourteen of these patients received highly active antiretroviral therapy (HAART) prior to the diagnosis of PML (*n* = 14/17; 82%). Median HIV-1 viral load at the time of diagnosis of PML was 17,500 copies/ml and ranged from 0 to 2,212,797 copies/ml. In two patients HIV-1 viral load was below the detectable range. Detailed information on HIV-1 viral load was not available for two patients. Immune status in patients with HIV/AIDS was generally poor with a median absolute CD4 T cell count of 70/μl (range 17–314/μl; norm: 300–2200/μl) and a median CD4/CD8 ratio of 0.12 (range 0.03–0.24, norm: 0.7- 2.8). In one patient CD4 T cell count data was missing.

Six patients had an underlying hematological or oncological malignancy (*n* = 6/37; 16%), of which one patient suffered from both, a solid tumor and a hematologic malignancy. Hematologic malignancies included acute myeloid leukemia (*n* = 1/37; 3%), chronic lymphoblastic leukemia (*n* = 1/37; 3%), IgG kappa myeloma (*n* = 1/37; 3%), follicular non-Hodgkin lymphoma (*n* = 2/37; 5%), and primary myelofibrosis (*n* = 1/37; 3%). The only solid tumor was a small cell bronchial carcinoma with pleural carcinomatosis (*n* = 1/37; 3%) and the patient also suffered from follicular non-Hodgkin lymphoma.

Two patients with underlying hematological malignancy underwent allogenic stem cell transplantation prior to the diagnosis of PML. One patient was diagnosed with definite PML two years after diagnosis of myelofibrosis and 20 months after allogenic stem cell transplantation. MRI scan and clinical symptoms were suggestive for PML. JCV was highly replicative with a VL of 17,408 copies/ml in CSF and 960 copies/ml in blood. The patient died two months after the diagnosis of PML. Another patient who suffered from acute myeloid leukemia also underwent allogenic stem cell transplantation eleven months prior to the diagnosis of definite PML. MRI scan showed typical signs of PML, JCV VL in CSF was 1,000 copies/ml confirmed the diagnosis. The patient had a fatal outcome and died within one month after the diagnosis of PML.

Five patients (*n* = 5/37; 14%) had a diagnosis of multiple sclerosis and received immunomodulatory treatment with natalizumab, a humanized mAb directed against the cell adhesion molecule α4-integrin (10, 11).

Another five patients (*n* = 5/37; 14%) suffered from sarcoidosis. In three patients (*n* = 3/5; 60%) diagnosis of sarcoidosis was confirmed by biopsy of mediastinal lymph node or lung showing non-caseating granulomas. One patient refused biopsy but radiological and clinical findings were typically for sarcoidosis (19). CD4 T cell counts were low in all patients where available (*n* = 3/5; 60%) (median 243/μl; range 101–270/μl, norm <500/μl). Soluble interleukin-2 receptor (sIL2R) was elevated in three of the five patients (*n* = 3/5; 60%) (median 713 U/ml; range 209 – 2182; norm <623 U/ml). Two patients never received immunosuppressive treatment for sarcoidosis before the diagnosis of PML (*n* = 2/5; 40%), one patient had not taken immunosuppressive medication for 10.5 years (*n* = 1/5; 20%), one patient received prednisolone in combination with azathioprine (*n* = 1/5; 20%) and another prednisolone in combination with methotrexate (*n* = 1/5; 20%).

Two patients (*n* = 2/37; 5%) had advanced liver cirrhosis, one of which had an underlying alpha-1 antitrypsin deficiency and reported chronic alcohol abuse. This patient underwent liver transplantation and received immunosuppressive treatment with tacrolimus for five years prior to the diagnosis of PML. One patient with liver cirrhosis was diagnosed with primary sclerosing cholangitis (PSC), autoimmune hepatitis (AIH) and ulcerative colitis as an underlying condition. Due to ulcerative colitis, this patient received immunosuppressive treatment with a combination of infliximab—a mAb directed against TNF-α–together with azathioprine and prednisolone.

One patient had mixed connective tissue disease (*n* = 1/37; 3%), which was treated with prednisolone and azathioprine. Another patient (*n* = 1/37; 3%) presented without any evident immunocompromised condition.

### Diagnosis of PML

We identified 44 patients with the diagnosis of PML using the patient register between January 2013 and August 2019 at the University Hospital Hamburg-Eppendorf, Germany. Patients were identified by appropriate ICD10 codes for PML.

In accordance with consensus statement criteria (13) review of the available medical records confirmed diagnosis of PML in 37 of 44 (84%) patients, with definite PML in 29 patients (*n* = 29/37; 78%), probable PML in four patients (*n* = 4/37; 11%), and four possible cases of PML (*n* = 4/37; 11%) (Figure 1).

Diagnosis of a definite PML was established based on typical clinical and radiographic presentation, in combination with JCV detection in CSF or classical histopathological findings in the brain biopsy. Quantitative JCV-PCR CSF analysis, available from *n* = 29/37 patients (78%), showed a viral load at the time of diagnosis between 20 and 400,000 JCV copies/ml with a median of 3,000 copies/ml.

**Figure 2:**
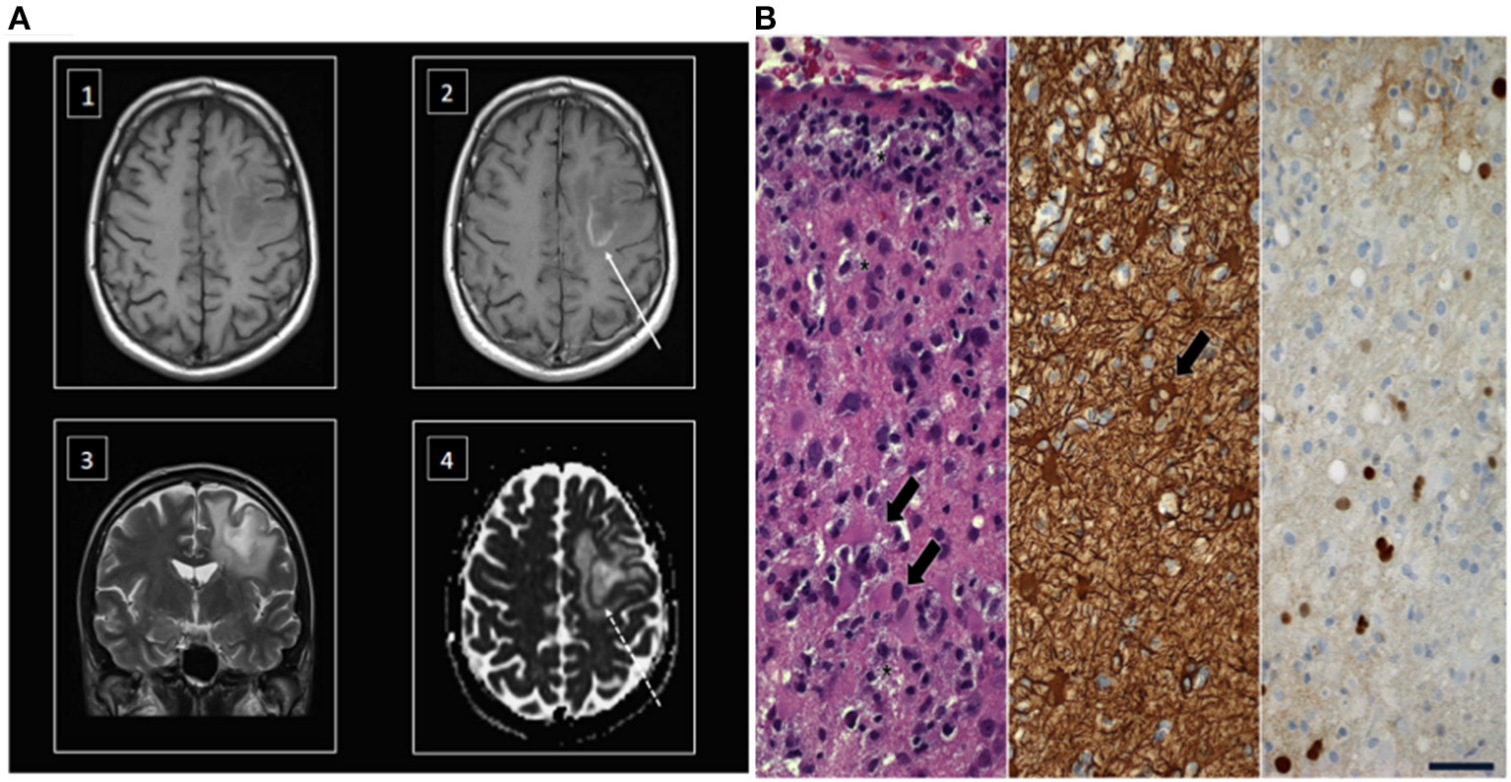
MRI scan and hispathology after stereotactic brain biopsy in a representative patient with PML. **(A)** Superatentional cPML lesion in an immunosuppressed patient with typical moderate mass effect in relation to size of the lesion. T1 weighted imaging before (1) and after contrast administration (2) showing a centrally hypointense lesion with a slightly hyperintense rim that enhances gadolinium (arrow) (3). The lesion typically involves the subcortical U-fibers in T2 weighted images (4). The ADC map of diffusion weighted imaging showing a restricted diffusion in the region of the enhancing ring (dotted arrow) and facilitated diffusion in the rest of the lesion. **(B)** Left. Stereotactic biopsy revealed active inflammation with demyelination. T cell infiltrates, numerous foamy macrophages (asterisks) and reactive gliosis (arrows); middle, immunohistochemical labeling of large reactive astrocytes (arrow) with antibodies against glial fibrillary acidic protein; right, immunohistochemical demonstration of SV40 protein in scattered cell nuclei; scale = 50 μm.

Four patients were diagnosed with a possible PML showing typical clinical and radiographic findings. Data on MRI scans with compatible radiological findings were available for 34 patients (*n* = 34/37; 92%) (Figure 2A).

Neuropathologic confirmation of JCV ascertained a definite PML diagnosis for *n* = 6/29 patients (20%). Figure 2B shows typical histopathological findings in a stereotactic biopsy comprising reactive gliosis with large astrocytes, foamy macrophages, and immunohistochemical detection of polyomavirus protein [simian virus 40 (SV40) protein] in a representative patient with ulcerative colitis and liver cirrhosis caused by PSC and AIH. T cell infiltrates as well as discrete gadolinium enhancement were indicative of an additional inflammatory reaction in this patient (inflammatory PML), which can be observed in some cases with the onset of immune reconstitution.

The most frequently observed clinical symptoms of PML were disorders in speech in 16 patients (43%), followed by cognitive and behavioral changes in 13 patients (35%), motor weakness in 13 patients (35%), unsteady gait in nine patients (24%), visual disorders in eight patients (22%), and seizures in only two patients (5%). All patients experienced more than one symptom.

Routine analysis of CSF was available in 92% of patients (*n* = 34/37). Abnormalities in CSF were found in *n* = 19/34 (56%) with elevated glucose levels in three patients (*n* = 3/19; 16%; median glucose 560 mg/L; range 470–1,060 mg/L; norm: 320–820 mg/L); elevated cell count in 15 patients (*n* = 15/19; 79%; median cell count 18 cells/μL; range 0–267/μL cells; norm: <15 cells/μL) and elevated protein levels in 13 patients (*n* = 13/19; 68%; median protein level 437 mg/L; range: 252–822 mg/L; norm: 140–500 mg/L).

### PML-Directed Treatment and Patient Outcome

Treatment attempts to improve the outcome of PML were documented in 13 patients (*n* = 13/37; 35%) (Table 2). Pharmacological agents included mirtazapine (*n* = 6/13; 46%), maraviroc (*n* = 2/13; 15%), immunoglobulins (*n* = 1/13; 8%), pembrolizumab (*n* = 1/13; 8%), and a combination of immunomodulatory agents (IL-7 and VP1-antigen immunization) (*n* = 1/13; 8%). One patient received combination therapy with immunoglobulins and mirtazapine (*n* = 1/13; 8%), another patient received a combination with maraviroc and mirtazapine (*n* = 1/13; 8%).

**Table 2:** Treatment attempts with PML directed treatment.

	**Total number of patients *n* (%)**	**Outcome**		
		**Death**	**Death**	**Stable diseases**
		**within 12 months** ***n*** **(%)**	**within 24 months** ***n*** **(%)**	***n*** **(%)**
**Initiation of PML directed treatment**	**13/37 (35)**	**4/13 (30)**	**5/13 (38)**	**6/13 (46)**
**Pharmacological agents used**				
Mirtazapine only	6/13 (46)	2/6 (33)	3/6 (50)	1/6 (16)
Immunomodulatory agents (IL-7 and VP1-Antigen Immunization)	1/13 (8)	0/1 (0)	0/1 (0)	1/1 (100)
Pembrolizumab (PD-1 inhibitor)	1/13 (8)	0/1 (0)	n.a.	1/1 (100)
Maraviroc only	2/13 (15)	0/2 (0)	0/2 (0)	2/2 (100)
Immunoglobulins	1/13 (8)	1/1 (100)	1/1 (100)	0/1 (0)
Combination of Maraviroc and Mirtazapine	1/13 (8)	1/1 (100)	1/1 (100)	0/1 (0)
Combination of Immunoglobulins and Mirtazapine	1/13 (8)	0/1 (0)	n.a.	1/1 (100)

Overall ten patients were lost to follow-up within a period of 24 months after diagnosis of PML (*n* = 10/37; 27%). Ten patients (*n* = 10/27; 37%) died within 12 months after diagnosis of PML, while another two patients died within 24 months after initial diagnosis (*n* = 12/27; 44%). Survival rates 12 and 24 months after diagnosis of PML were 63 and 56%, respectively. While patients with HIV/AIDS and autoimmune diseases showed the best survival rates (76 and 80%, respectively), patients with hematological malignancies had the lowest survival rates (20%) (Table 1). In the group of patients with sarcoidosis, only one patient died within two months after the diagnosis of PML (*n* = 1/5; 20%).

## Discussion

The prevalence of PML in the general population is estimated to be low. To develop PML, patients generally present with suppressed cellular immunity as a predisposing factor. Thus, distinct populations at risk are described in the literature. Although combined antiretroviral therapy (cART) significantly decreased the incidence of PML, HIV-positive individuals are still the most affected population (20).

The second-largest population at risk is patients with various forms of hematological malignancies. In recent times, PML may also be an adverse effect of immunosuppressive therapies with mAbs, especially natalizumab, frequently used in patients with relapsing-remitting multiple sclerosis (21–23). Unlike natalizumab, until today no cases of PML have been reported in association with vedolizumab, a recently developed mAb to α4β7 integrin, which is approved for the treatment of active inflammatory bowel disease.

In our observational monocenter study, the majority of PML cases were associated with HIV/AIDS (46%) and patients receiving mAbs (22%)—in the majority of cases natalizumab—followed by patients with hematological malignancies (16%). In line with the literature, no patient developed PML after the use of vedolizumab.

Notably, five out of 37 patients (13.5%) were diagnosed with sarcoidosis as an underlying disease. So far this has only been described in few case reports (24–28) and should therefore be considered to be a relevant risk group.

Previous German cohort studies focused on patients with natalizumab-associated PML, with two of them focusing rather on immunological aspects of PML (29–31).

Early diagnosis of PML is critical because a prompt attempt to restore the patient's immune status currently represents the only option to prevent the rapid deterioration of neurological status and improve survival rates (32, 33).

Clinically, it is often challenging to establish the diagnosis of PML due to the broad spectrum of presented neurologic signs and symptoms. PML may affect multiple areas of the brain and often causes multifocal lesions (15, 34). The most common clinical features are motor weakness, cognitive dysfunction, ataxia, visual symptoms, and/ or speech disorders (13, 20, 35). Our data revealed a similar distribution of first neurological symptoms with 43% showing speech disturbances, followed by cognitive, and behavioral changes in 35% and motor symptoms in 35% of patients.

In addition to clinical neurological symptoms, typical radiological findings in brain MRI, as well as laboratory diagnostics may support the diagnosis of PML. In accordance with the AAN diagnostic criteria, it is necessary to evaluate clinical and imaging findings together with the detection of JCV DNA in the brain (13). As replication of JCV DNA takes place in CNS-resident oligodendrocytes, the absence of detection of JCV DNA in CSF does not rule out PML. Still, 78% of the patients presented here showed positive detection of JCV DNA in CSF. Furthermore, 20% of patients showed typical histopathological findings after stereotactic brain biopsy, confirming definite PML (Figure 2B).

In accordance with the consensus statement criteria (13) four cases of possible PML were included in this study. The diagnosis of possible PML in these patients was based on typical clinical and MRI findings as assessed by our board certified neuroradiology attendings at our high volume university medical center. Of note, MRI findings are often unreliable and depend on the radiologist's experience and expertise and specialized neuro radiologists are not always available at all centers and have to be evaluated in the context of the variable and heterologous neurologic signs and symptoms of patients with severe underlying co-morbidities. Our current real-world cohort illustrates the difficulties and challenges clinicians are still facing when establishing the diagnosis of PML.

In CSF analysis normal to slightly increased cell counts are known to be associated with PML. However, the underlying diseases such as HIV/ AIDS or the use of natalizumab can significantly influence cell counts in CSF and thus makes it challenging to interpret results correctly (36, 37).

In our cohort 19 patients showed abnormalities in the CSF with elevated cell counts in 15 patients (*n* = 15/19; 79%). In line with published data, abnormalities were mild and cell counts ranged from 0 to 267 cells/μL (38).

Cellular responses mediated by CD4 and CD8 T cells play an essential role in controlling JCV disease. CD4 T cell deficit is a significant risk factor for PML, particularly a CD4 T cell count of <200 cells/μL. In line with this, 18 patients (out of 24 patients with available CD4 T cell counts) had a CD4 T cell count <250/μl at the time of diagnosis of PML in our study. Nonetheless, only a fraction of (HIV-1) patients with low CD4 T cell counts develops PML, and two PML patients in our cohort had normal CD4 T cell counts. This indicates that additional functional defects may facilitate PML development, which currently are not understood nor routinely measured.

None of the presented patients showed evident signs of PML associated IRIS. If the patient survives, PML-IRIS is almost always present in some form, even though the manifestation is often missed without systematic follow-up.

At present, no specific prophylaxis or effective anti-JCV treatment exists. Therefore, patient outcome entirely depends on an individual's ability to restore immune function in response to JCV. Potential pharmacological substances for PML-directed treatment such as antiviral agents, immune response modulators, and immunization strategies are experimentally used (14). Due to the devastating prognosis, in our cohort, 35% of patients received therapy with PML-directed agents based on compassionate use.

The serotonergic receptor 5HT2AR has been described as a receptor for JCV cell entry into human glial cells. Targeting the serotonin receptor has shown anti-JCV activity *in vitro* (39). The most frequently used drug in our study population was the serotonin receptor blocker mirtazapine in 46% of patients (*n* = 6/13), of whom only one patient showed stable diseases, while three patients died within 24 months after diagnosis of PML.

Maraviroc, a C–C chemokine receptor type 5 (CCR5) antagonist that blocks CCR5-mediated inflammation that is approved for the treatment of CCR5-tropic HIV infection (40), has also been used as a treatment approach for PML associated IRIS (14, 41). However, in 2014, there was one report of a patient in whom maraviroc failed to control PML-associated IRIS (42). Three of our patients received maraviroc (*n* = 3/13; 23%), one in combination with mirtazapine (43). In all patients, a stable course of PML was reported.

The checkpoint inhibitor pembrolizumab, targeting programmed cell death protein 1 (PD-1) is approved for different unresectable or metastatic solid tumors in Europe. PD-1 expression is known to be up-regulated on lymphocytes and JCV–specific CD8 T cells in patients with PML (44). Recently, Cortese et al. published data of seven PML patients that were treated with pembrolizumab, which was administered on a compassionate-use basis. The authors described a clinical improvement or stabilization in five patients and concluded that the PD-1 blockade represents a potential treatment option for PML (45). Our data cautiously support these findings with one patient showing stable disease following pembrolizumab therapy assessed 12 months after the initial diagnosis of PML. In contrast, in 2019 Pawlitzki et al. described an ultimately fatal case of PML after a treatment attempt with pembrolizumab. The reasons for this treatment failure are not clear at this point and this case underlines the need to prospectively evaluate the efficacy of PML treatment with pembrolizumab in further controlled studies (46).

One patient received a vaccine consisting of a CD4+ T cell epitope of the recombinant JCV major capsid protein VP1 together with recombinant human IL-7 to further boost JCV-specific T-cell responses. The details of this case and another patient were reported in 2014 (47). The vaccination resulted in a production of neutralizing antibodies against JCV, which correlated with resolution of PML progression in brain MRI, induction of robust JCV VP-1 specific CD4 T cell proliferation, substantial reductions in JCV DNA viral load in CSF, and showed an overall improvement of clinical symptoms (47).

Despite all specific treatment efforts, PML remains a significant concern for individuals with compromised cellular immune responses and is associated with poor survival. In the present cohort, we assessed survival rates 12 and 24 months after diagnosis of PML (63 vs. 56%, respectively), with the lowest survival rates in patients with hematological malignancies (20%) and the highest survival rates in patients with HIV/AIDS and autoimmune diseases (Table 1). These findings are in line with the current literature, where survival rates range from 40 to 50% in the first months after diagnosis (48). Very poor survival has been described for patients with hematological malignancies (49), while the survival of patients with iatrogenic immunosuppression improved due to the option of cessation of the causing immunosuppressive agent (33). Nonetheless, the poor prognosis due to the underlying hematological malignancy itself compared to other causes of PML has to be taken into account when considering these survival rates.

Overall, patients treated with experimental agents such as the IL-7 and VP1-Antigen Immunization, pembrolizumab and maraviroc only (*n* = 4) showed better survival (100% respectively) compared to patients treated with mirtazapine only (*n* = 6), whom of only one patient survived and showed stable diseases after 24 months of follow-up. Concluding higher efficacy of these experimental drugs is not possible due to the very small number of patients and retrospective design of this study. Currently, experimental treatment strategies comprise antiviral agents, immune modulators, and immunization strategies and should ideally address prophylaxis and treatment of PML. Only few prospective studies for new therapeutics for PML are ongoing or completed (50). The small patient number and importantly the lack of good animal models for PML mostly limit existing studies in this context. Further basic research and clinical studies are urgently needed (50).

In conclusion, our results emphasize that despite improved molecular biological detection and modern radiological procedures, the diagnosis of PML remains challenging. Although PML is rare, with increasing availability and use of mAbs all physicians should be aware of and sensitized to a potential PML risk. As no effective PML-directed treatment is available, early detection and screening for risk factors is essential to prevent irreversible neurological consequences and death in patients with PML.

## Data Availability Statement

The raw data supporting the conclusions of this article will be made available by the authors, without undue reservation.

## Ethics Statement

The local Ethics Committee of the Ä*rztekammer Hamburg* approved the study (WF-179/20).

## Author Contributions

LG analyzed the patient data. CH performed the histological examination of the brain. EG analyzed the patients' MRI scans. SR, SJ, and MF were the patients' doctors in charge. AH, MA, and JS reviewed the literature and made an important contribution to revising the manuscript. CB analyzed the patient data, reviewed the literature, and drafted the paper. CB and JS designed and directed the project. All authors contributed to the article and approved the submitted version.

## Conflict of Interest

The authors declare that the research was conducted in the absence of any commercial or financial relationships that could be construed as a potential conflict of interest.
